# From ciliary sulcus asymmetry to vault prediction: an hSTS-based approach to individualized sizing of implantable collamer lenses in myopic eyes

**DOI:** 10.3389/fmed.2026.1762551

**Published:** 2026-02-26

**Authors:** Zeyu Zhu, Miao Diao, Chunhao Fu, Wanling Wu, Guiying Zhang, Jingyuan Tian, Chuchu Zhong, Lingling Zhong, Ying Zhu, Jun Li

**Affiliations:** 1 Dalian Medical University, Dalian, China; 2 He Eye Specialist Hospital, Shenyang, China; 3 He University, Shenyang, China; 4 College of Physics and Communication Electronics, Jiangxi Normal University, Nanchang, China; 5 Fuzhou Experimental School, Fuzhou, China; 6 Department of Ophthalmology, Dalian Third People’s Hospital Affiliated to Dalian University of Technology, Dalian, Liaoning, China; 7 Dalian Municipal Eye Hospital, Dalian, Liaoning, China; 8 Liaoning Provincial Key Laboratory of Cornea and Ocular Surface Diseases, Dalian, Liaoning, China; 9 Technology Engineering Research Center, Dalian, Liaoning, China

**Keywords:** implantable collamer lens (ICL), myopia, personalized surgery, sulcus-to-sulcus, ultrasound biomicroscopy (UBM), vault

## Abstract

**Objective:**

To investigate the distribution and correlations of horizontal and vertical sulcus-to-sulcus diameters (hSTS/vSTS) in myopic eyes with different refractive errors, and to develop a predictive model for postoperative vault after posterior chamber phakic implantable collamer lens (ICL) implantation based on key anterior segment parameters.

**Methods:**

This retrospective study included two non-overlapping cohorts of myopic patients who underwent ICL implantation at a single tertiary eye hospital. The morphology cohort comprised 244 eyes, classified into moderate, high, and extreme myopia groups according to spherical equivalent (SE). Ultrasound biomicroscopy (UBM) was used to measure hSTS and vSTS; inter-group comparisons and correlation analyses were performed. The model development cohort included 44 patients (72 eyes). In addition to STS, preoperative anterior segment parameters (corneal curvature, horizontal corneal diameter, anterior chamber depth, lens thickness, and axial length) were recorded, and ICL size was selected according to the STAAR nomogram. One month postoperatively, the vault was measured using anterior segment optical coherence tomography. Univariate correlation and stepwise multiple linear regression were applied to identify factors and derive a prediction formula.

**Results:**

The ciliary sulcus was predominantly vertically elliptical in the overall population (vSTS > hSTS). In the extreme myopia group, both hSTS and vSTS were significantly larger than in moderate and high myopia groups, with a greater vSTS-hSTS difference (0.63 ± 0.38 mm, all *P* < 0.05). SE correlated weakly and negatively with hSTS (*r* = –0.177, *P* = 0.006) and vSTS (*r* = –0.243, *P* < 0.001), while hSTS and vSTS were strongly positively correlated (*r* = 0.781, *P* < 0.001). In the modeling cohort, stepwise regression identified hSTS and ICL size as independent predictors of vault (adjusted *R*^2^ = 0.657), yielding the formula: vault (μm) = –2286.216 - 164.416 × hSTS (mm) + 375.487 × ICL size.

**Conclusion:**

The higher myopic refractive error is associated with a more pronounced vertically elliptical ciliary sulcus. Routine measurement of both vertical and horizontal STS, combined with a vault prediction model based on hSTS and ICL size, may facilitate individualized ICL sizing, improve vault predictability, and enhance surgical safety.

## 1 Introduction

Myopia is the most common refractive error worldwide, and its prevalence continues to rise, making it a major public health concern (1). For adult myopic patients who are not suitable candidates for corneal laser surgery, posterior chamber phakic implantable collamer lens (ICL) implantation has become one of the mainstream refractive options because of its excellent visual quality, predictability, and long-term safety (2, 3).

Postoperative vault is a key determinant of the safety of ICL implantation (4). Insufficient vault may lead to contact between the ICL and the anterior capsule of the crystalline lens, increasing the risk of anterior subcapsular opacification and cataract, whereas excessive vault may push the iris forward, impair aqueous outflow, and trigger intraocular pressure elevation or even pupillary block (5, 6). The central hole in the V4c design has been shown to relatively reduce some of these risks, but it does not eliminate these risks entirely (7, 8). Furthermore, the inherent optical design of the V4c lens, characterized by a flat anterior surface and increased edge thickness, particularly in high myopic corrections, can elevate the risks of angle crowding, pigment dispersion, and potential endothelial touch (7, 9).

Vault is largely determined by the match between ICL size and the patient’s intraocular anatomy, among which the sulcus-to-sulcus (STS) distance is a critical parameter (9). In current clinical practice, ICL sizing is still mainly guided by the STAAR company nomogram based on external measurements such as horizontal corneal diameter (white-to-white, WTW) and anterior chamber depth (ACD) (9, 10). However, previous studies have shown that the correlation between WTW and the true STS distance is limited, which may contribute to suboptimal postoperative vault (11, 12).

Ultrasound biomicroscopy (UBM) can clearly visualize the ciliary body and ciliary sulcus and is regarded as the gold standard for measuring STS (13). Several studies have reported that incorporating UBM-derived STS into ICL sizing can improve the predictability of postoperative vault (9, 14). Notably, the ciliary sulcus is not perfectly circular in most eyes, but tends to be vertically elliptical, with the vertical STS (vSTS) exceeding the horizontal STS (hSTS) (15). This anatomical asymmetry may vary with refractive status and directly influence ICL stability and postoperative vault (9, 15). Nevertheless, most existing studies have been limited to a single refractive error range or have focused only on hSTS, and systematic data on the distribution and differences of hSTS and vSTS across different myopia severities remain scarce (7, 9).

In addition, although UBM provides STS measurements that more closely reflect the true intraocular anatomy, to effectively integrate UBM-derived STS and other anterior segment parameters into preoperative planning and construct quantitative, accurate vault prediction models that offer intuitive decision support for ICL sizing still requires in-depth research. Therefore, this study aimed to comprehensively investigate the distribution and correlations of hSTS, vSTS, and their differences in myopic eyes with varying refractive errors, and to develop and preliminarily evaluate a regression model for predicting postoperative ICL vault based on key anterior segment parameters, with particular emphasis on STS, in order to provide new strategies for individualized ICL sizing and enhanced surgical safety.

## 2 Materials and methods

### 2.1 Research subject

This retrospective clinical study included myopic patients who underwent ICL V4c implantation at He Eye Specialist Hospital (Shenyang, China) between June 2021 and November 2024. Two parallel, non-overlapping cohorts were constructed from patients operated on during this period.

The morphology cohort: A total of 122 myopic patients (244 eyes) were enrolled, including 39 men (78 eyes) and 83 women (166 eyes), with a mean age of 26.15 ± 7.53 years. Based on preoperative SE, eyes were classified into a moderate myopia group (–6.0D < SE ≤ –3.0D, 45 eyes), a high myopia group (–10.0D < SE ≤ –6.0D, 150 eyes), and an extreme myopia group (SE ≤–10.0D, 49 eyes). UBM was performed on all eyes to measure hSTS and vSTS. This part of the study was approved by the Human Subject Ethics Subcommittee of Shenyang He Eye Specialist Hospital [approval No. IRB(2025)K012.01].

The model-development cohort: To construct the vault prediction model, an independent sample of 44 patients (72 eyes) was selected from the same time period. The mean age in this cohort was 25.72 ± 6.13 years. ICL size in all eyes was determined according to the STAAR company nomogram. This part of the study was approved by the Human Subject Ethics Subcommittee of Shenyang He Eye Specialist Hospital [approval No. IRB(2024)K002.01].

The study adhered to the tenets of the Declaration of Helsinki, and written informed consent was obtained from all participants before surgery.

### 2.2 Inclusion and exclusion criteria

Inclusion criteria: (1) age ≥ 18 years; (2) diagnosis of myopia or myopic astigmatism and scheduled for ICL V4c implantation; (3) ACD ≥ 2.8 mm; (4) corneal endothelial cell density (ECD) ≥ 2,000 cells/mm^2^ (5) relatively stable refraction for at least 2 years (change ≤ 0.50 D per year); (6) preoperative intraocular pressure (IOP) between 10 and 21 mmHg; (7) willingness to undergo ICL implantation and ability to provide written informed consent.

Exclusion criteria: (1) history of ocular surgery; (2) presence of ocular diseases other than simple refractive error that could affect visual function, such as glaucoma, keratoconus, uveitis, or other significant pathology; (3) unstable refractive status; (4) severe or uncontrolled systemic diseases, such as diabetes mellitus, hypertension, thyroid dysfunction, or autoimmune diseases; (5) severe psychological or psychiatric disorders (e.g., anxiety, depression) that could interfere with perioperative cooperation or follow-up compliance.

### 2.3 Preoperative examination and measurements

All enrolled patients underwent a comprehensive preoperative evaluation, including uncorrected visual acuity (UCVA), best-corrected visual acuity (BCVA), subjective refraction, IOP measurement, slit-lamp biomicroscopy, fundus examination, ocular surface assessment, corneal endothelial cell density, and B-scan ultrasonography, to rule out contraindications to ICL implantation.

#### 2.3.1 Common preoperative parameters and STS measurement

ACD and ECD were measured in all included eyes. STS was assessed using a 50-MHz UBM (MD-300L, Maida, China) by the same experienced examiner. During the examination, a scleral shell was placed and filled with coupling medium, and patients were instructed to fixate on an external target. Radial scans were obtained along the horizontal and vertical meridians, and hSTS (3–9 o’clock) and vSTS (6–12 o’clock) were manually measured. Each meridian was measured three times, and the mean value was used for analysis.

#### 2.3.2 Additional parameters in the model-development cohort

In the model-development cohort, additional anterior segment parameters were recorded. An optical interferometric biometer (AL-Scan, NIDEK, Japan) was used to measure axial length (AL) and lens thickness. Anterior segment analysis was performed with a Scheimpflug camera system (Pentacam HR, OCULUS, Germany) to obtain the flat and steep keratometric powers (K1, K2), WTW, ACD, anterior chamber angle (ACA), and anterior chamber volume (ACV). STS measurements with UBM were performed as described above. These parameters, together with the postoperative vault, were used to construct the vault prediction model.

### 2.4 Surgical procedure

EVO ICL (V4c) sphere, cylinder, and size were selected preoperatively using the manufacturer’s OCOS nomogram based on age, refraction, keratometry, ACD, and WTW. All surgeries were performed by the same experienced surgeon. After adequate pharmacologic mydriasis and topical anesthesia, a 3.0-mm clear corneal main incision was created, and the ICL was inserted into the posterior chamber. The four footplates were positioned in the ciliary sulcus, with the lens centered. At the end of surgery, the ophthalmic viscosurgical device was thoroughly removed, and the incision was hydrated to ensure watertight closure.

### 2.5 Postoperative evaluation

Postoperatively, visual acuity, IOP, and ECD were routinely monitored. In the model-development cohort, particular attention was paid to the ICL vault at 1 month after surgery. Vault was measured using a spectral-domain optical coherence tomography system (Cirrus HD-OCT 6000, Carl Zeiss Meditec Inc., United States) in its anterior segment imaging mode. The built-in software was used to determine the distance between the ICL and the anterior crystalline lens capsule automatically.

### 2.6 Statistical analysis

Statistical analyses were performed using SPSS 26.0 (IBM Corp., NY, United States). Continuous variables with a normal distribution were expressed as mean ± standard deviation, and non-normally distributed data were expressed as median and interquartile range *M(Q1, Q3)*. Categorical variables were presented as counts and percentages *n* (%).

In the morphology cohort, continuous variables were compared among refractive groups using one-way analysis of variance, with LSD tests for pairwise comparisons. Categorical variables were compared using the χ^2^-test. Pearson correlation analysis was used to assess the relationships between SE and STS parameters. In the model-development cohort, Pearson or Spearman correlation analysis was applied, as appropriate, to evaluate associations between preoperative parameters and postoperative vault. Stepwise multiple linear regression was performed with vault as the dependent variable to construct the prediction model. *P* < 0.05 was considered statistically significant.

## 3 Results

### 3.1 Baseline characteristics of the morphology cohort

A total of 244 eyes were included in the morphology cohort, comprising 78 eyes (32.0%) from male patients and 166 eyes (68.0%) from female patients; right and left eyes were evenly distributed (122 eyes each). The age of the patients ranged from 18 to 49 years. The mean preoperative SE was –8.16 ± 2.53 D. The mean hSTS and vSTS were 11.92 ± 0.43 mm and 12.45 ± 0.46 mm, respectively, with a mean vSTS-hSTS difference of 0.53 ± 0.29 mm and an hSTS/vSTS ratio of 95.74% ± 2.24%. Other preoperative parameters are summarized in Table 1.

**TABLE 1:** Baseline morphologic characteristics of the ciliary sulcus.

Parameter	Overall (*N* = 244 eyes)
Age (years)	26.15 ± 7.53
Sex (male/female, eyes)	78 / 166
Laterality (right/left, eyes)	122 / 122
SE (D)	–8.16 ± 2.53
Sphere (D)	–7.54 ± 2.44
Cylinder (D)	–1.24 ± 0.95
AL(mm)	26.54 ± 1.36
ACD (mm)	3.19 ± 0.28
ECD (cells/mm^2^)	2995.76 ± 296.26
hSTS (mm)	11.92 ± 0.43
vSTS (mm)	12.45 ± 0.46
vSTS-hSTS (mm)	0.53 ± 0.29
hSTS/vSTS (%)	95.74 ± 2.24

SE, spherical equivalent; AL, axial length; ACD, anterior chamber depth; ECD, Endothelial Cell Density; hSTS, horizontal sulcus-to-sulcus diameter; vSTS, vertical sulcus-to-sulcus diameter; vSTS-hSTS, difference between vertical and horizontal STS diameters; hSTS/vSTS, ratio of horizontal to vertical STS diameters.

### 3.2 Biometric and anatomical characteristics among different refractive groups

Based on SE, eyes were classified into moderate, high, and extreme myopia groups. Sphere, cylinder, axial length, and ciliary sulcus diameters are summarized for the three different refractive groups. Both sphere and AL increased significantly and progressively from the moderate to the extreme myopia group (both *P* < 0.001). The magnitude of the cylinder also differed significantly among groups (*P* < 0.001).

Within each group, vSTS was significantly greater than hSTS (all *P* < 0.001). Between-group comparisons showed that both hSTS and vSTS were significantly larger in the extreme myopia group than in the moderate and high myopia groups (all *P* < 0.05) (Table 2).

**TABLE 2:** Baseline characteristics and ciliary sulcus dimensions among different refractive groups.

Group	n	Sphere (D)	Cylinder (D)	AL(mm)	hSTS(mm)	vSTS(mm)	vSTS vs. hSTS	
							*t*	*P*
Moderate myopia group	45	–4.12 ± 0.94	–0.87 ± 0.79	25.19 ± 0.89	11.74 ± 0.44	12.24 ± 0.46	–11.46	< 0.001*
High myopia group	150	–7.46 ± 1.16^a^	–1.19 ± 0.94^a^	26.47 ± 0.95^a^	11.92 ± 0.42^a^	12.44 ± 0.45^a^	–24.64	< 0.001*
Extreme myopia group	49	–10.93 ± 1.61^ab^	–1.73 ± 0.92^ab^	28.00 ± 1.40^ab^	12.08 ± 0.39^ab^	12.71 ± 0.39^ab^	–11.46	< 0.001*
*F*		361.66	10.96	85.71	7.95	13.95	–	–
*P*		< 0.001*	<0.001*	< 0.001*	<0.001*	< 0.001*	–	–

Within-group comparisons between hSTS and vSTS were performed using paired sample *t*-test. Between-group comparisons of continuous variables were performed using a one-way ANOVA with LSD tests for *post-hoc* pairwise comparisons.

^a^*P* < 0.05 versus the moderate myopia group;

^b^*P* < 0.05 versus the high myopia group. *P < 0.05.

### 3.3 Between-group comparison of directional STS asymmetry

One-way ANOVA showed a significant difference in the vSTS-hSTS difference among the three refractive groups (*F* = 3.05, *P* = 0.049). *Post-hoc* pairwise comparisons revealed that the vSTS-hSTS difference was greater in the extreme myopia group than in both the moderate and high myopia groups (both *P* < 0.05), whereas no significant difference was found between the moderate and high myopia groups. In contrast, the hSTS/vSTS ratio did not differ significantly among the three groups (*P* = 0.10) (Table 3).

**TABLE 3:** Comparison of directional STS asymmetry among different refractive groups.

Group	*N*	vSTS-hSTS (mm)	hSTS/vSTS (%)
Moderate myopia group	45	0.50 ± 0.29	0.96 ± 0.02
High myopia group	150	0.52 ± 0.26	0.96 ± 0.02
Extreme myopia group	49	0.63 ± 0.38^ab^	0.95 ± 0.03
Test statistics		*F* = 3.05, *P* = 0.049*	*F* = 2.32, *P* = 0.100

Between-group comparisons were performed using one-way ANOVA, with LSD tests for *post-hoc* pairwise comparisons.

^a^*P* < 0.05 versus the moderate myopia group; ^b^*P* < 0.05 versus the high myopia group. *P < 0.05.

### 3.4 Correlations between SE, hSTS, and vSTS

**FIGURE 1:**
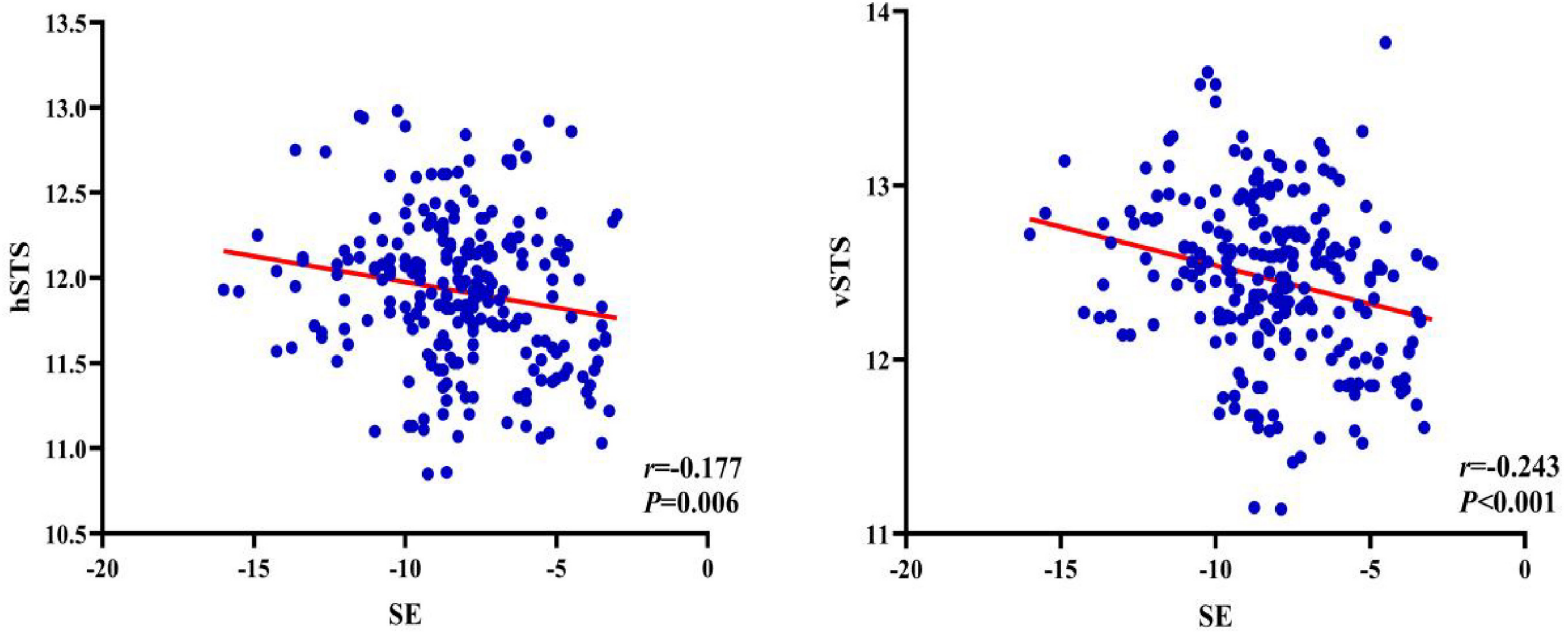
Correlation between SE and hSTS as well as vSTS.

**FIGURE 2:**
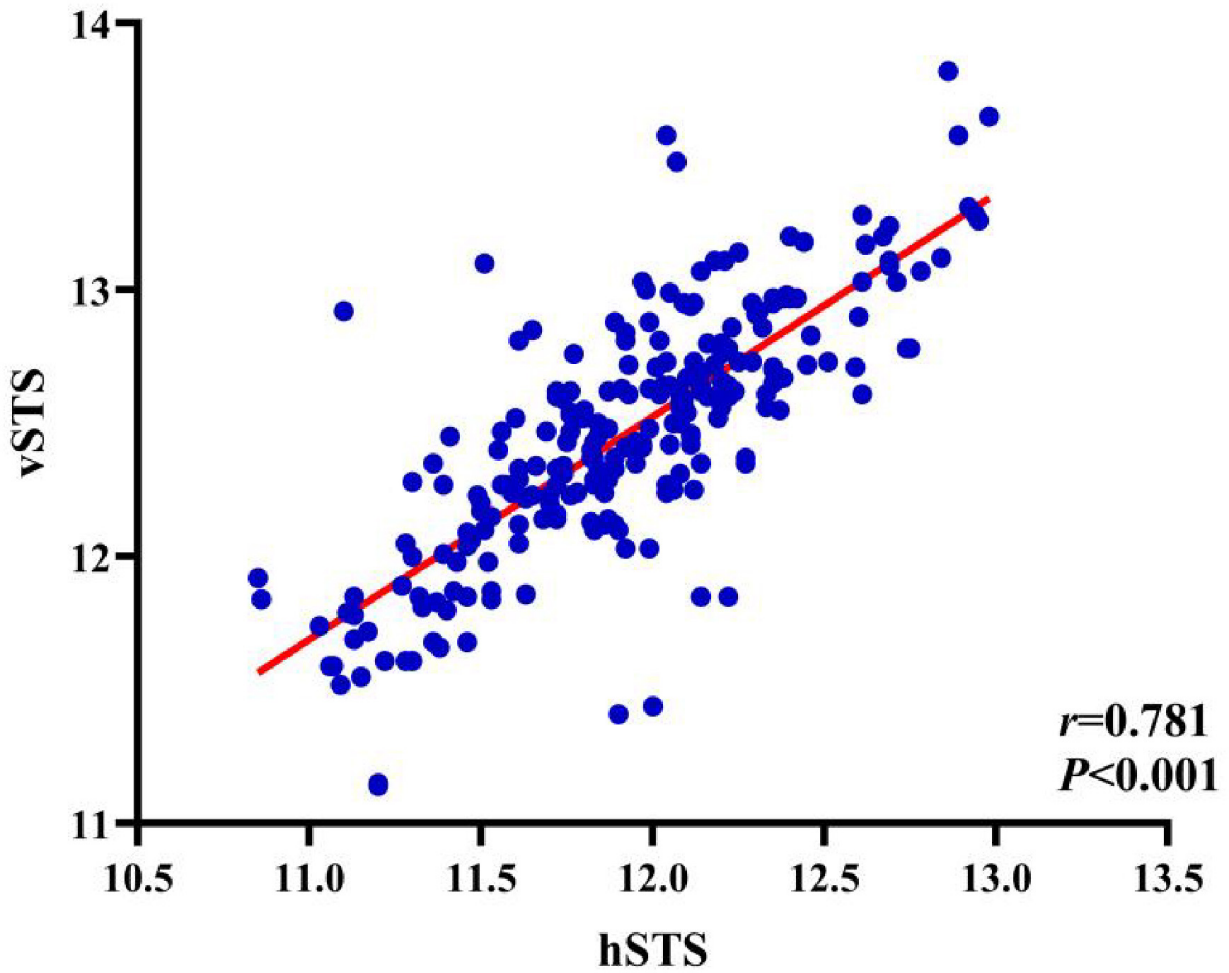
Correlation between hSTS and vSTS.

Pearson correlation analysis showed that SE was negatively correlated with both hSTS and vSTS (*r* = -0.18, *P* = 0.006; *r* = -0.24, *P* < 0.001, respectively), although the strength of these associations was weak. In contrast, hSTS and vSTS were strongly positively correlated (*r* = 0.78, *P* < 0.001) (Figures 1, 2 and Table 4).

**TABLE 4:** Correlation analysis among SE, hSTS, and vSTS.

Variable	SE	hSTS	vSTS
	*r* (P-value)	*r* (P-value)	*r* (P-value)
SE	—	–0.18 (0.006*)	–0.24 ( < 0.001*)
hSTS	–0.18 (0.006*)	—	0.78 ( < 0.001*)
vSTS	–0.24 ( < 0.001*)	0.78 ( < 0.001*)	—

Values are Pearson correlation coefficients (*r*) with corresponding P-values in parentheses. *P < 0.05.

### 3.5 Distribution patterns and shifting trends of hSTS and vSTS

**FIGURE 3:**
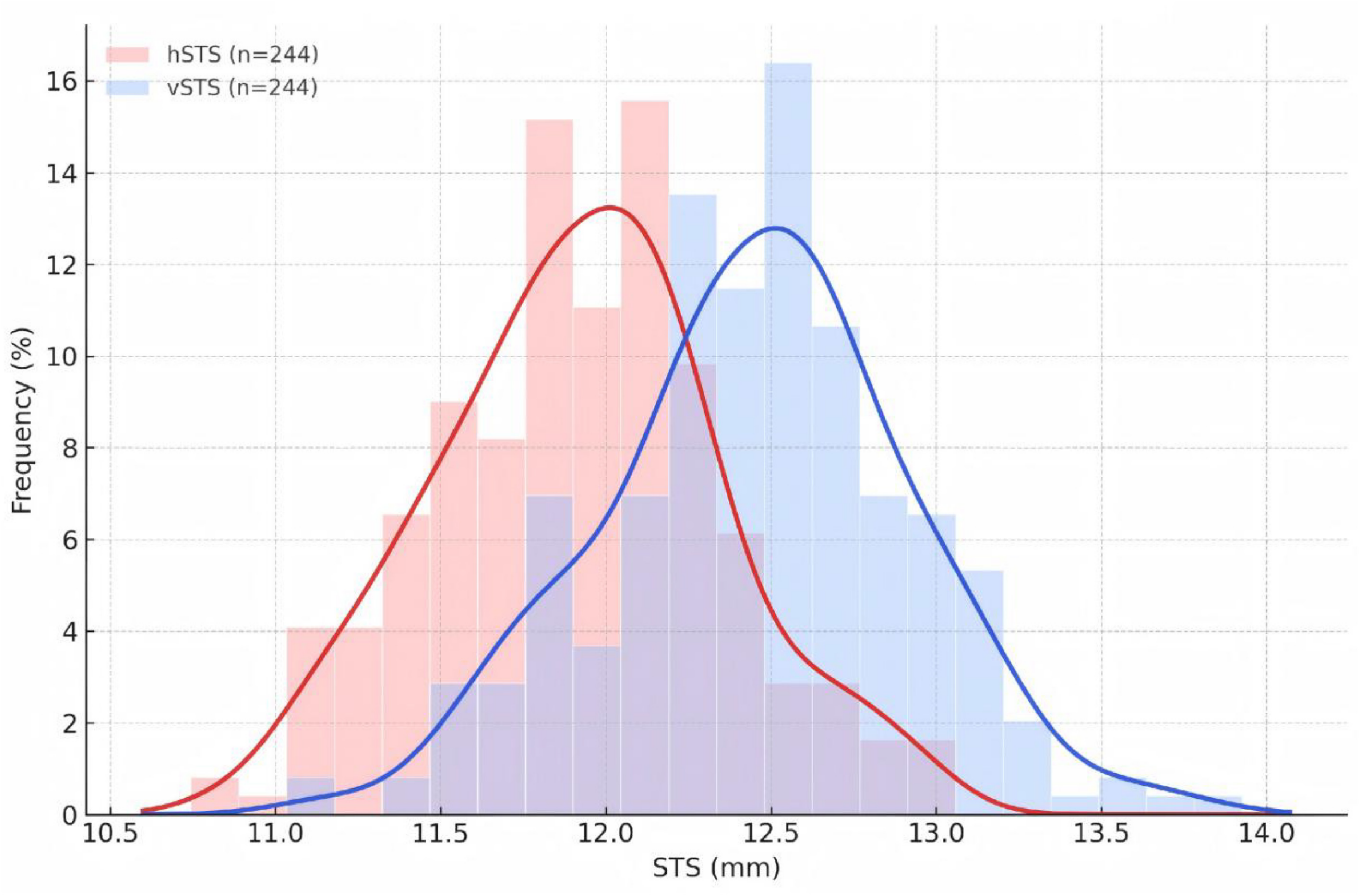
Overlaid distributions of hSTS and vSTS.

**FIGURE 4:**
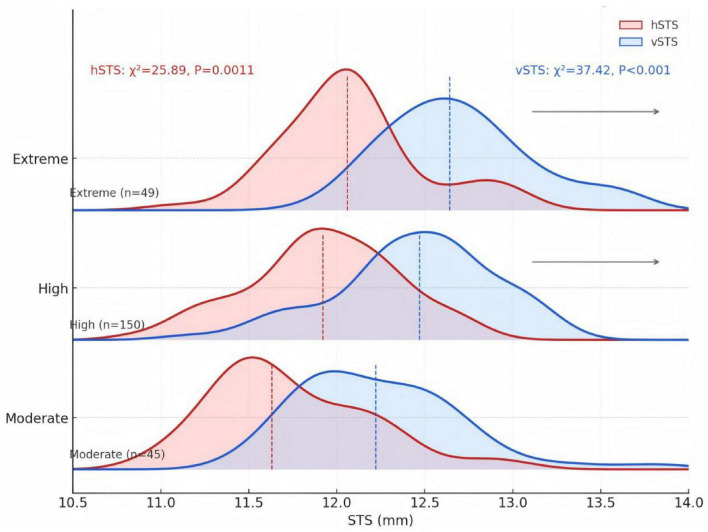
Ridgeline densities of hSTS and vSTS across refractive groups. The vertical dashed line within each density area indicates the median (50th percentile, P50) value. The overall distribution differences between groups were statistically significant.

In the morphology cohort, hSTS and vSTS were most frequently distributed in the 11.50–12.00 mm (38.6%) and 12.50–13.00 mm (37.4%) ranges, respectively (Figures 3, 4 and Table 5). With increasing SE, both hSTS and vSTS showed a significant shift toward larger diameter ranges (all *P* < 0.001), and the extreme myopia group exhibited higher proportions in the larger intervals (all *P* < 0.05). These findings indicate that higher myopia is associated not only with increased mean hSTS and vSTS values, but also with a global shift of their overall distribution toward larger diameters.

**TABLE 5:** Distribution of hSTS and vSTS in different refractive groups.

Diameter range (mm)	Overall (*n* = 244), %	Moderate myopia group (*n* = 45), %	High myopia group (*n* = 150), %	Extreme myopia group (*n* = 49), %
**hSTS**				
10.50–11.00	0.8	0	1.3	0
11.00–11.50	16.8	35.6	16.0^a^	2.0^ab^
11.50–12.00	38.6	37.8	40	34.7
12.00–12.50	34.8	22.2	34	49.0^a^
12.50–13.00	9	4.4	8.7	14.3
Test statistics	χ^2^ = 25.89, *P* = 0.001			
**vSTS**				
11.00–11.50	1.6	0	2.7	0
11.50–12.00	14.3	35.6	12.7^a^	0.0^ab^
12.00–12.50	36.1	35.6	36.7	34.7
12.50–13.00	37.4	24.4	38	46.9
13.00–13.50	9	2.2	10	12.2
13.50–14.00	1.6	2.2	0	6.1^b^
Test statistics	χ^2^ = 37.42, *P* < 0.001			

Between-group comparisons of the distribution of hSTS and vSTS were performed using the χ^2^-test, with Bonferroni-corrected χ^2^-tests for *post-hoc* pairwise comparisons.

^a^*P* < 0.05 versus the moderate myopia group;

^b^*P* < 0.05 versus the high myopia group.

### 3.6 Distribution characteristics of vSTS-hSTS and the hSTS/vSTS ratio

**FIGURE 5:**
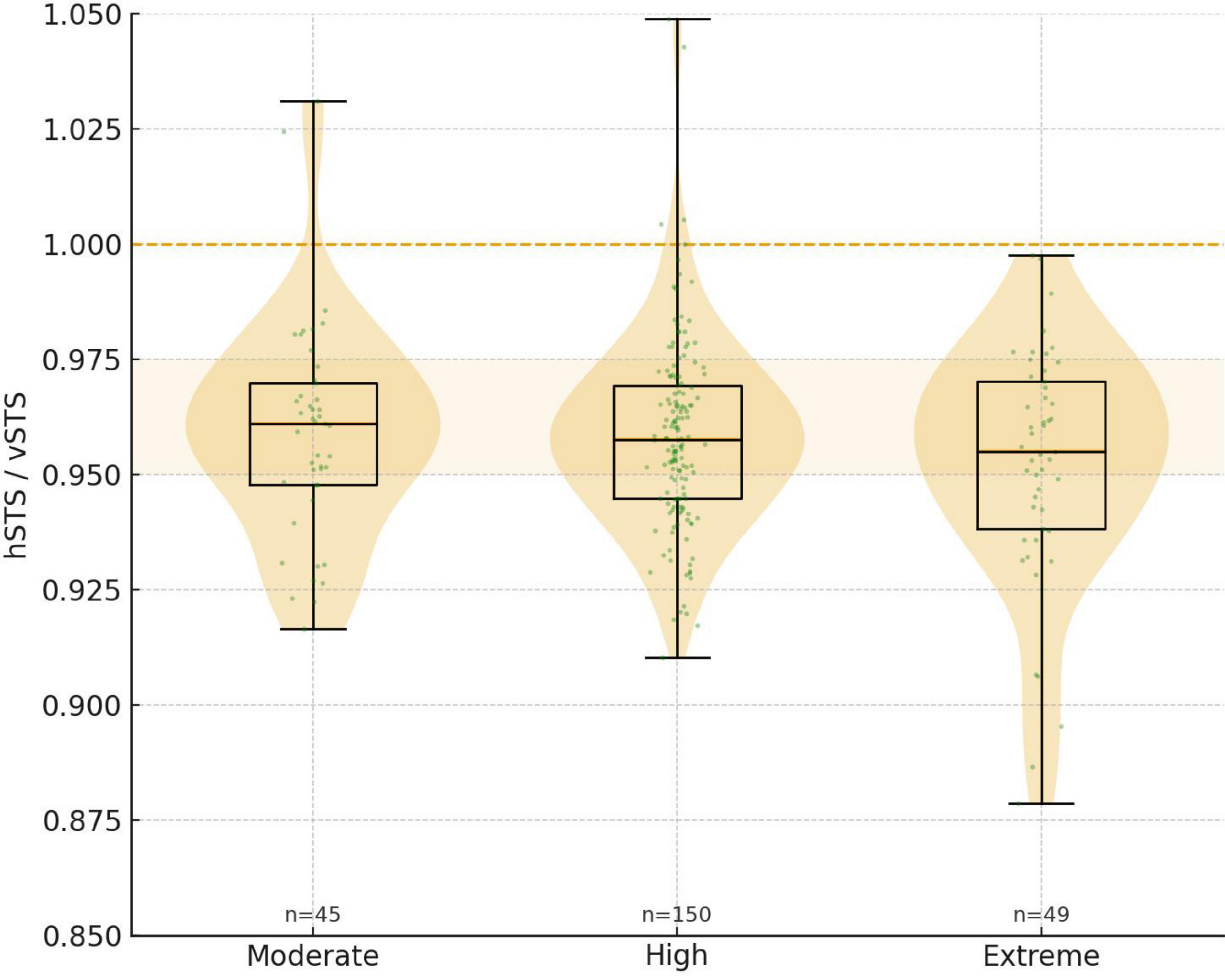
Violin plot of group-wise distribution of hSTS/vSTS.

In the morphology cohort, the vSTS-hSTS difference was most frequently distributed in the 0.50–1.00 mm range (48.4%). The proportion of eyes with vSTS-hSTS ≥ 0.50 mm increased with higher SE, being 44.4, 56.0, and 57.1% in the moderate, high, and extreme myopia groups, respectively. Chi-square analysis showed a significant difference in the distribution of vSTS-hSTS among the three refractive groups (χ^2^ = 19.003, *P* = 0.040). Notably, different values > 1.50 mm were observed only in the extreme myopia group. In contrast, the distribution of the hSTS/vSTS ratio did not differ significantly among the three myopia groups (χ^2^ = 17.470, *P* = 0.232). However, in the vast majority of eyes, this ratio was < 1.0, with proportions of 95.6, 96.7, and 100.0% in the moderate, high, and extreme myopia groups, respectively (Figure 5 and Table 6).

**TABLE 6:** Distribution of vSTS-hSTS differences and hSTS/vSTS ratios among different refractive groups (%).

Index	Overall (*n* = 244), %	Moderate myopia group (*n* = 45), %	High myopia group (*n* = 150), %	Extreme myopia group (*n* = 49), %
**vSTS-hSTS (mm)**				
–1.00 to –0.50	0.4	0	0.7	0
–0.50 to 0.00	2.4	4.4	2.7	0
0.00–0.50	44.7	51.1	42	46.9
0.50–1.00	48.4	42.2	52.7	40.8
1.00–1.50	2.9	2.2	2	6.1
1.50–2.00	1.2	0	0	6.1^ab^
Test statistic	χ^2^ = 19.003, *P* = 0.040*			
**hSTS/vSTS ratio**				
0.850–0.875	0.4	0	0	2
0.875–0.900	1.3	0	0	6.1
0.900–0.925	4.5	6.7	4	4.1
0.925–0.950	26.2	22.2	27.3	26.5
0.950–0.975	50.8	51.1	52.7	44.9
0.975–1.000	14.3	15.6	13.3	16.3
1.000–1.025	1.2	2.2	1.3	0
1.025–1.050	1.2	2.2	1.3	0
Test statistic	χ^2^ = 17.470, *P* = 0.232			

Between-group comparisons of the distribution of hSTS and vSTS were performed using the χ^2^-test, with Bonferroni-corrected χ^2^*-t*ests for *post-hoc* pairwise comparisons. ^a^*P* < 0.05 versus the moderate myopia group; ^b^*P* < 0.05 versus the high myopia group.

### 3.7 Baseline characteristics of the vault-modeling cohort

A total of 44 patients (72 eyes) were included in the vault-modeling cohort. The mean age was 25.72 ± 6.13 years, and ICL size selection followed the STAAR Nomogram. Preoperative biometric parameters and 1-month postoperative vault measurements are summarized in Table 7.

**TABLE 7:** Baseline characteristics of the vault-modeling cohort.

Parament	Mean ± SD
Age (years)	25.72 ± 6.13
K1 (D)	43.08 ± 1.45
K2 (D)	44.49 ± 1.60
WTW (mm)	11.66 ± 0.41
ACD (mm)	3.19 ± 0.22
ACA (°)	40.03 ± 4.26
ACV (mm^2^)	199.46 ± 29.08
LT (mm)	3.70 ± 0.25
AL (mm)	26.38 ± 1.29
hSTS (mm)	11.86 ± 0.35
vSTS (mm)	12.45 ± 0.37
Vault (μm)	581.04 ± 140.13

K1, flat keratometry; K2, steep keratometry; WTW, white-to-white; ACD, anterior chamber depth; ACA, anterior chamber angle; ACV, anterior chamber volume; LT, lens thickness; AL, axial length; hSTS, horizontal sulcus-to-sulcus diameter; vSTS, vertical sulcus-to-sulcus diameter.

### 3.8 Univariate correlations between preoperative parameters and postoperative vault.

Univariate correlation analysis at 1 month postoperatively showed that corneal flat and steep keratometric powers (K1, K2) were negatively correlated with vault (*r* = -0.502 and -0.444, respectively; both *P* < 0.05). In contrast, WTW, AL, hSTS, vSTS, and ICL size were positively correlated with vault (*r* = 0.383, 0.546, 0.546, 0.542, and 0.782, respectively; all *P* < 0.05). No significant correlations were found between vault and ACD, ACA, ACV, or LT (all *P* > 0.05) (Table 8).

**TABLE 8:** Correlations between 1-month postoperative vault and preoperative parameters.

Parameter	Correlation coefficient (*r*)	*P*-value
K1 (D)	-0.502	< 0.05*
K2 (D)	-0.444	< 0.05*
WTW (mm)	0.383	< 0.05*
AL (mm)	0.546	< 0.05*
hSTS (mm)	0.546	< 0.05*
vSTS (mm)	0.542	< 0.05*
ICL size (mm)	0.782	< 0.05*
ACD (mm)	0.062	0.636
ACA (°)	-0.212	0.077
ACV (mm^2^)	0.239	0.051
LT (mm)	-0.102	0.46

Pearson’s correlation analysis was used; values in the table represent correlation coefficients (*r*). K1, flat keratometry; K2, steep keratometry; WTW, white-to-white; ACD, anterior chamber depth; ACA, anterior chamber angle; ACV, anterior chamber volume; LT, lens thickness; AL, axial length. *P < 0.05.

### 3.9 Stepwise multiple linear regression analysis

Stepwise multiple linear regression was performed with 1-month postoperative vault as the dependent variable and the parameters that were significant in univariate analysis (K1, K2, WTW, AL, hSTS, vSTS, and ICL size) as candidate predictors. In the final model, only ICL size (standardized β = 1.135, *P* < 0.001) and hSTS (standardized β = –0.413, *P* = 0.002) remained as independent predictors of vault. The resulting regression equation was:


Vault⁢at⁢ 1⁢month⁢(μ⁢m)=-2286.216-164.416×hSTS⁢(mm)+375.487×ICLsize⁢(mm) (1)

The model showed good goodness-of-fit (*R*^2^ = 0.667, adjusted *R*^2^ = 0.657), and all variance inflation factors (VIF) were < 5, indicating no relevant multicollinearity and a stable model (Table 9).

**TABLE 9:** Preoperative parameters independently associated with 1-month postoperative vault.

Preoperative parameter	Unstandardized coefficient (B)	Standardized coefficient (β)	*P*	VIF
hSTS	–164.416	–0.413	0.002*	3.493
ICL size	375.487	1.135	<0.001*	3.493

Stepwise multiple linear regression was applied; *P < 0.05.

## 4 Discussion

ICL postoperative vault is a key determinant of safety after ICL implantation. Its magnitude depends largely on the match between ICL size and the patient’s intraocular anatomy and is shaped by a complex interaction of multiple factors (9, 16). Achieving precise preoperative selection, therefore, requires detailed anterior segment information that corresponds to the actual attachment site of the ICL footplates (5, 14). With respect to key parameters, standard clinical AS-OCT can provide several useful parameters, such as corneal curvature and ACD, but it cannot consistently and reliably visualize structures posterior to the iris for ciliary sulcus assessment in a routine clinical setting (12, 17). In contrast, UBM affords direct visualization of the ciliary body and sulcus and enables precise measurement of STS distances, providing a more dependable basis for evaluating ICL sizing and positioning (14). However, although a vertically elliptical ciliary sulcus configuration has been reported previously (18), the refractive-status–dependent changes in STS asymmetry have not been systematically characterized, which, to some extent, has limited the wider adoption of STS-based approaches for ICL sizing and vault prediction. Ultimately, the safety and efficacy of ICL surgery hinge on achieving an optimal biomechanical match between the lens and the posterior chamber space (9). This fundamental requirement underscores the enduring necessity for precise assessment of the ciliary sulcus anatomy, regardless of evolving ICL lens design (19, 20).

Our study employed SE for group stratification, as it is the principal clinical determinant of myopia severity in refractive surgery (21, 22). Although AL is a more direct geometric biometric, its use in clinical grouping lacks standardized thresholds (23–25). In the ciliary sulcus morphology cohort, UBM measurements were performed using a standardized protocol recommended by expert consensus in a relatively large sample of myopic eyes, and STS diameters were analyzed in fine 0.50-mm intervals. This approach systematically demonstrated that a vertically elliptical sulcus configuration (vSTS > hSTS) is ubiquitous in myopic eyes, consistent with previous studies in pseudophakic eyes (18), and further showed that this asymmetry becomes significantly more pronounced with increasing myopic SE. Accordingly, the diameter ratio hSTS/vSTS was further incorporated into the analysis, and the proportion of eyes with hSTS/vSTS < 1 was found to reach 100% in the extreme myopia group, providing morphologic confirmation that the degree of vertical ellipticity escalates as myopia worsens. These findings are consistent with previous reports and quantitatively supplement the description of this progressive pattern (1). In addition, the results from this morphology cohort suggest that the influence of SE on STS may involve a “dual mechanism.” First, a “global enlargement” effect was identified, in which both hSTS and vSTS increased as SE became more myopic, consistent with the axial length–based findings reported by Wang et al. (26) Second, a “progressive asymmetry” was observed, characterized by a greater vSTS-hSTS difference and a higher proportion of eyes with vSTS-hSTS ≥ 0.5 mm. Taken together, these observations indicate that the ciliary sulcus undergoes increasingly marked vertical elliptical remodeling as myopia deepens. Consequently, preoperative ICL planning should account for the differential impact of refractive status on horizontal and vertical STS, which is important for selecting lens size, preventing postoperative rotation, and maintaining a stable vault. These morphologic findings not only complement previous work on the spatial structure of the STS but also provide a clear anatomical basis for understanding the mechanical behavior of the ICL and the accompanying changes in vault after implantation.

Building on these morphologic findings, an independent vault-modeling cohort was used to develop a predictive model in which preoperative hSTS and ICL size were entered into a multiple linear regression with 1-month postoperative vault as the dependent variable. The model demonstrated good performance in a single-center dataset, with a simple structure, easily obtainable input parameters, and intuitively interpretable output. Compared with more complex machine-learning approaches that require large training datasets, this conventional regression model is more convenient to implement at the current sample size while still providing stable and reliable predictions, thereby enhancing its potential for clinical translation and wider application.

In clinical practice, ICL size is still most commonly selected on the basis of WTW and ACD (7, 27). However, several studies have shown that the correlation between WTW and STS is limited, and acceptable predictive accuracy is achieved only when the difference between the two lies within a narrow range; overreliance on WTW may therefore lead to inappropriate ICL sizing and abnormal postoperative vault (28). At the same time, previous reports have consistently suggested that ACD is closely associated with postoperative vault (7, 9, 10). In this study, however, once hSTS was included in the stepwise regression model, the independent associations of WTW and ACD with vault were no longer significant. This finding indicates that, within an STS-based sizing strategy, hSTS more directly reflects the key anatomical determinants of postoperative vault, whereas conventional anterior segment parameters derived from corneal and anterior chamber geometry make a relatively attenuated independent contribution.

In addition to WTW and ACD, the vault-modeling cohort also evaluated the contribution of other preoperative parameters, including corneal curvature and AL. Univariate analyses showed that K1 and K2 were moderately negatively correlated with postoperative vault, and AL was also associated with vault, suggesting that corneal shape and AL may participate in vault formation by altering anterior segment configuration. These observations are broadly consistent with previous reports indicating that corneal curvature and AL may influence vault primarily through their impact on ACD (23). However, in the multivariable regression analysis, the effect sizes of these parameters were relatively small, and their explanatory power for vault was clearly inferior to that of hSTS and ICL size; therefore, they were not retained in the final model. Likewise, vSTS was not included in the regression equation, which may reflect the fact that, in current clinical practice, ICLs are predominantly implanted along or near the horizontal meridian, and postoperative vault is thus mainly constrained by hSTS (9, 15). Future studies should investigate different implantation axes and rotational states to comprehensively characterize the relationships among hSTS, vSTS, vault, and ICL stability, with the aim of further refining STS-based individualized sizing and axis-planning strategies.

This study has several limitations. First, it was a single-center retrospective analysis conducted exclusively with the ICL V4c model, and both cohorts had relatively modest sample sizes; as a result, the external validity of the findings is limited, and they should be interpreted with caution until confirmed in larger, multicenter prospective studies. In the ciliary sulcus morphology cohort, refractive status was classified primarily on the basis of SE; AL and corneal curvature were not incorporated into the grouping scheme, nor was a distinction made between spherical and cylindrical components. Moreover, measurements were restricted to horizontal and vertical meridional STS and did not cover other STS orientations and adjacent structures such as the ciliary processes. So the depiction of the spatial structure of the STS still needs to be supplemented. In the vault-modeling cohort, the consistency of UBM measurements has not been systematically verified, and potential inter-device conformity among different UBM systems from various manufacturers has not been evaluated. In addition, the set of anterior segment parameters entered into the model remained relatively limited, without inclusion of finer anatomical indices such as iris thickness and ciliary process morphology. Furthermore, parameters describing the anterior curvature of the crystalline lens, such as crystalline lens rise (CLR), which may refine vault prediction (29), were not included in our analysis and represent an important direction for model optimization. Although ACD no longer showed an independent association with vault once hSTS was introduced into the multivariable model, the present data are insufficient to conclude that its role can be completely replaced by STS. Future multicenter studies with larger samples and a more comprehensive panel of anterior segment parameters, together with external validation, are needed to improve further the accuracy and robustness of STS-based vault prediction models.

## 5 Conclusion

This study confirmed that the ciliary sulcus in myopic eyes is predominantly vertically elliptical, and that the vSTS-hSTS difference increases with higher myopic SE, suggesting the need to routinely assess both horizontal and vertical STS—rather than relying solely on WTW or a single horizontal diameter—when planning ICL surgery in cases of high and extreme myopia. Based on this understanding, a simple vault prediction model incorporating hSTS and ICL size was constructed using readily obtainable parameters, and it may serve as a useful complement to existing sizing nomograms, providing a practical reference for individualized ICL selection and vault management in highly myopic patients.

## Data Availability

The data analyzed in this study is subject to the following licenses/restrictions: Data will be made available on request. Requests to access these datasets should be directed to Jun Li, robin_lijun@sina.com.
